# Supplementary material for: A unified neural account of contextual and individual differences in altruism
Source: eLife. 2023 Feb 8;12:e80667. doi: 10.7554/eLife.80667 (PMC9908080; doi:10.7554/eLife.80667)
Supplement: Supplementary file 1. [file elife-80667-supp1.docx]

**Table S1.** **Generalized linear mixed-effects model results of choice data.**

|  | Model 1 | | Model 2 | | Model 3 | | |
| --- | --- | --- | --- | --- | --- | --- | --- |
| Fixed effects | Estimate  (95% CI) | p-value | Estimate  (95% CI) | p-value | Estimate  (95% CI) | p-value | |
| Intercept | -0.03  (-0.13 – 0.07) | 0.57 | -0.06  (-0.16 – 0.04) | 0.22 | -0.02  (-0.05 – 0.01) | | 0.20 |
| $\Delta S$ | 3.77  (3.65 – 3.89) | < 0.001 | 3.70  (3.59 – 3.82) | < 0.001 | - | - | |
| $\Delta O$ | 0.56  (0.51 – 0.61) | < 0.001 | 0.55  (0.51 – 0.60) | < 0.001 | - | - | |
| CON | 0.12  (0.06 – 0.19) | < 0.001 | 0.05  (-0.004 – 0.11) | 0.07 | - | - | |
| $\Delta S$ *$\Delta O$ | 0.13  (0.01 – 0.24) | 0.03 | - | - | - | - | |
| $\Delta S$ *CON | -0.35  (-0.47 – -0.23) | < 0.001 | - | - | - | - | |
| $\Delta O$ *CON | -0.02  (-0.07 – 0.03) | 0.50 | - | - | - | - | |
| $\Delta S$ *$\Delta O$ *CON | 0.23  (0.11 – 0.35) | < 0.001 | - | - | - | - | |
| df | 15,447 | |  | |  | | |
| LL | -3796 | | -3828 | | -10737 | | |
| BIC | 7679 | | 7704 | | 21494 | | |

$\Delta S$, self-payoff change between the 2^nd^ and 1^st^ option; $\Delta O$, other-payoff change between the 2^nd^ and 1^st^ option; CON, context; CI, confidence interval; df, degree of freedom; LL, log-likelihood; BIC, Bayesian Information Criterion
