# Supplementary material for: A unified neural account of contextual and individual differences in altruism
Source: eLife. 2023 Feb 8;12:e80667. doi: 10.7554/eLife.80667 (PMC9908080; doi:10.7554/eLife.80667)
Supplement: Supplementary file 2. [file elife-80667-supp2.docx]

**Table S2.** **Generalized linear mixed-effects model results of choice data show that the presentation order (i.e., 1^st^ or 2^nd^) of the more equal or unequal option does not bias individuals’ choices.**

| Fixed effects | Estimate | 95% CI | z-value | p-value |
| --- | --- | --- | --- | --- |
| Intercept | - 1.07 | -1.35 – -0.79 | -7.41 | < 0.001 |
| $\Delta O$ | 4.83 | 4.61 – 5.05 | 43.65 | < 0.001 |
| CON | -6.32 | -6.60 – -6.04 | -44.58 | < 0.001 |
| Ind_ref | 0.06 | -0.03 – 0.15 | 1.30 | 0.193 |
| $\Delta O$*CON | 0.79 | 0.57– 1.01 | 7.14 | < 0.001 |
| df | 15,486 | | | |
| LL | -5813 | | | |
| BIC | 11684 | | | |

$\Delta S$, self-payoff change between the 2nd and 1st option; $\Delta O$, other-payoff change between the 2nd and 1st option; CON, context; Ind_ref, indicator of the presentation order for the more equal option; CI, confidence interval;df, degree of freedom; LL, log-likelihood; BIC, Bayesian Information Criterion
