# Supplementary material for: A unified neural account of contextual and individual differences in altruism
Source: eLife. 2023 Feb 8;12:e80667. doi: 10.7554/eLife.80667 (PMC9908080; doi:10.7554/eLife.80667)
Supplement: Supplementary file 3. [file elife-80667-supp3.docx]

**Table S3.** **Linear mixed-effects model results of RT data.**

| Fixed effects | Estimate | 95% CI | t-value | p-value |
| --- | --- | --- | --- | --- |
| Intercept | 0.79 | 0.72 – 0.86 | 21.98 | < 0.001 |
| \|$\Delta S$ \| | -0.09 | -0.11 – -0.07 | -7.44 | < 0.001 |
| \|$\Delta O$ \| | -0.03 | -0.05 – -0.003 | -2.19 | 0.03 |
| CON | -0.03 | -0.04 – -0.02 | -4.28 | < 0.001 |
| \|$\Delta S$ \|*\|$\Delta O$ \| | -0.004 | -0.02 – 0.01 | -0.49 | 0.62 |
| \|$\Delta S$ \|*CON | 0.04 | 0.02 – 0.06 | 3.31 | < 0.001 |
| \|$\Delta O$ \|*CON | 0.03 | 0.01 – 0.06 | 2.81 | 0.005 |
| \|$\Delta S$ \|*\|$\Delta O$ \|*CON | -0.01 | -0.02 – 0.004 | -1.45 | 0.15 |
| df | 15,447 | | | |
| LL | -4184 | | | |
| BIC | 8464 | | | |

$\Delta S$, self-payoff change between the 2^nd^ and 1^st^ option; $\Delta O$, other-payoff change between the 2^nd^ and 1^st^ option; CON, context; CI, confidence interval;df, degree of freedom; LL, log-likelihood; BIC, Bayesian Information Criterion
