# Supplementary material for: A unified neural account of contextual and individual differences in altruism
Source: eLife. 2023 Feb 8;12:e80667. doi: 10.7554/eLife.80667 (PMC9908080; doi:10.7554/eLife.80667)
Supplement: Supplementary file 4. [file elife-80667-supp4.docx]

**Table S4.** **Bounds of OU parameters.**

| Parameters | Lower bound | Upper bound |
| --- | --- | --- |
| $\alpha$ (Decision threshold) | 0.6 | 3 |
| $\beta$ (Starting point) | -2 | 2 |
| $\kappa$ (Drift rate) | -1 | 1 |
| $\omega$ (Weight on others) | -1 | 1 |
| $\lambda$ (leak strength) | -2 | 2 |
| $\tau$ (non-decision time) | 0.01 | 1 |
