# Supplementary material for: A unified neural account of contextual and individual differences in altruism
Source: eLife. 2023 Feb 8;12:e80667. doi: 10.7554/eLife.80667 (PMC9908080; doi:10.7554/eLife.80667)
Supplement: Supplementary file 5. [file elife-80667-supp5.docx]

**Table S5. Model comparison results.**

| Model | Parameters | BIC (Mean ± SE) |
| --- | --- | --- |
| OU model (full) | $\alpha_{\left( c, s \right)}$, $\beta_{\left( c, s \right)}$, $\kappa_{\left( c, s \right)}$, $\omega_{\left( c, s \right)}$, $\lambda_{\left( c, s \right)}$, $\tau_{\left( c, s \right)}$ | 4058 ± 46 |
| DDM | $\alpha_{\left( c, s \right)}$, $\beta_{\left( c, s \right)}$, $\kappa_{\left( c, s \right)}$, $\omega_{\left( c, s \right)}$, $\tau_{\left( c, s \right)}$ | 4136 ± 48 |
| OU model (fixed $\omega$) | $\alpha_{\left( c, s \right)}$, $\beta_{\left( c, s \right)}$, $\kappa_{\left( c, s \right)}$, $\omega_{\left( s \right)}$, $\lambda_{\left( c, s \right)}$, $\tau_{\left( c, s \right)}$ | 4127 ± 45 |
| OU model (fixed $\alpha$) | $\alpha_{\left( s \right)}$, $\beta_{\left( c, s \right)}$, $\kappa_{\left( c, s \right)}$, $\omega_{\left( c, s \right)}$, $\lambda_{\left( c, s \right)}$, $\tau_{\left( c, s \right)}$ | 4133 ± 46 |
| OU model (fixed $\omega\& \alpha$) | $\alpha_{\left( s \right)}$, $\beta_{\left( c, s \right)}$, $\kappa_{\left( c, s \right)}$, $\omega_{\left( s \right)}$, $\lambda_{\left( c, s \right)}$, $\tau_{\left( c, s \right)}$ | 4117 ± 46 |

$\alpha_{\left( c, s \right)}$, decision threshold; $\beta_{\left( c, s \right)}$, starting point; $\kappa_{\left( c, s \right)}$, drift rate modulator; $\omega_{\left( c, s \right)}$, relative weight on others’ payoffs; $\lambda_{\left( c, s \right)}$, leak strength; $\tau_{\left( c, s \right)}$, non-decision time (nDT); c for conditions (c = DIS for disadvantageous inequality context, c = ADV for advantageous inequality context), s for participants (s = 1, ..., N_participants_). In the last three models, $\alpha_{\left( s \right)}$ and $\omega_{\left( s \right)}$ assumed the same decision threshold and/or weight on others’ payoff across contexts for each participant. SE, standard error.
